# Progression Analysis and Stage Discovery in Continuous Physiological Processes Using Image Computing

**DOI:** 10.1155/2010/107036

**Published:** 2010-05-26

**Authors:** Lior Shamir, Salim Rahimi, Nikita Orlov, Luigi Ferrucci, Ilya G Goldberg

**Affiliations:** 1 Laboratory of Genetics, National Institute on Aging, National Institutes of Health, 251 Bayview Boulevard, Baltimore,MD 21224, USA; 2 Clinical Research Branch, National Institute on Aging, National Institutes of Health, 3001 Hanover Street, Baltimore,MD 21225, USA

## Abstract

We propose an image computing-based method for quantitative analysis of continuous physiological processes that can be sensed by medical imaging and demonstrate its application to the analysis of morphological alterations of the bone structure, which correlate with the progression of osteoarthritis (OA). The purpose of the analysis is to quantitatively estimate OA progression in a fashion that can assist in understanding the pathophysiology of the disease. Ultimately, the texture analysis will be able to provide an alternative OA scoring method, which can potentially reflect the progression of the disease in a more direct fashion compared to the existing clinically utilized classification schemes based on radiology. This method can be useful not just for studying the nature of OA, but also for developing and testing the effect of drugs and treatments. While in this paper we demonstrate the application of the method to osteoarthritis, its generality makes it suitable for the analysis of other progressive clinical conditions that can be diagnosed and prognosed by using medical imaging.

## 1. Introduction

Osteoarthritis (OA) is a highly prevalent chronic clinical condition that limits mobility and causes substantial disability in late life [1]. It is estimated that  of the population over the age of 65 have radiographic evidence of osteoarthritis [2], and given the increasing longevity in the industrialized world the prevalence of osteoarthritis is expected to increase further in the developed countries. While OA is one of the most prevalent diseases in the industrialized world, the physiological mechanisms of OA are poorly understood [3]. Yet, due to the increasing prevalence of knee osteoarthritis and its consequent effects on functional limitation and general life quality at older ages, there is a growing need for scientific tools that can be reliably used to study the mechanisms of OA.

**Figure 1:**
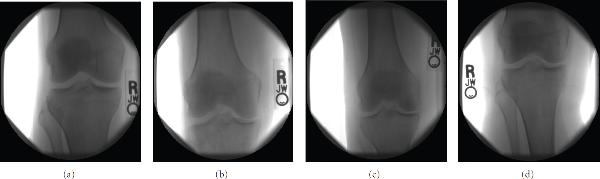
**X-ray images of four different KL grades: (a) 0 (normal), (b) 1 (doubtful), (c) 2 (mild), (d) 3 (moderate)**.

The presence and progression of osteoarthritis is usually evaluated by trained radiologists, who read knee X-rays and score them by using the standard Kellgren-Lawrence (KL) system [4, 5]. The KL classification scheme is a validated method for classifying individual joints into one of five grades, with 0 representing healthy joints, 1 representing*doubtful* OA, 2 representing*mild* OA, 3*moderate* OA, and 4 being the most severe radiographic disease. This classification is based on features of*osteophytes* (bony growths adjacent to the joint space),*narrowing* of part or all of the tibial-femoral joint space, and*sclerosis* of the subchondral bone, which reflect the progression of the disease. Figure 1 shows four knee X-rays of KL grades 0 (normal), 1 (doubtful), 2 (mild), and 3 (moderate).

It should be noted that while the Kellgren-Lawrence classification is the most commonly used classification scheme, there is no scientific evidence that the KL system provides an accurate direct assessment of the progression of OA [6, 7]. This downside of the KL classification scheme limits its potential as an objective tool that can be used to directly study the mechanisms and nature of OA, as well as assess the efficacy of drugs and treatments.

Another downside of the KL scoring system is that the parameters used for the classification are not discrete, and therefore different readers may have different assessments of the progression of each parameter, leading to a different conclusion regarding the presence or severity of [8, 9]. This subjectiveness makes the KL scores rough approximations of the actual progress of the disease. Although the KL scale has just five degrees of severity, even well-trained and highly experienced readers assign the same score to the same knee X-ray in just 80% of the cases [6, 10].

An alternative way to diagnose and evaluate the progression of OA is by applying quantitative image analysis methods that can measure subtle changes in the bone structure, which have been correlated with biochemical, biomechanical, and structural alterations of the articular cartilage and the subchondral bone tissues [11, 12] associated with cartilage degeneration [13, 14]. Previous studies have shown that the alteration of the bone structure can be sensed by analyzing features of the radiographic bone texture, and can be used to detect osteoarthritis by using image computing methods [10, 15–20].

While these previous studies used the radiographic bone texture for the purpose of automatic detection of osteoarthritis, the high prevalence of OA introduced an emerging need for clinical and scientific tools that can provide an objective and quantitative measurement of OA progression. These tools can be used for understanding the mechanisms of the disease and ultimately for studying the effect of drugs and treatments that may be proposed in the future and will require testing and evaluation. In this study we propose a method that applies image computing to quantitative measurement of the progression of OA and show how this method can be used to study the nature of the disease and potentially be used to better understand the mechanisms that are associated with cartilage degeneration. The analysis revealed little difference between healthy and*doubtful* osteoarthritic knees (KL grade 0 and KL grade 1); a finding that could be confirmed by using images obtained with a different imaging technique, MRI. This showed that the presented technique is sensitive to changes in the disease state of an individual knee, regardless of imaging technique used to analyze the joint in question. We argue that the multipurpose nature of the method can potentially make it a useful tool for studying other progressive clinical conditions that can be diagnosed or assessed by using imaging.

## 2. Methods

The image analysis method used in this study is*wndchrm* [21, 22], which has been found effective for the analysis and detection of osteoarthritis using knee X-rays [10].*Wndchrm* is a multipurpose image classification method that makes use of a large set of image features, extracted from several image transforms and compound transforms. The image features used by*wndchrm* can be classified into high-contrast features, textures (e.g., Haralick, Tamura), statistical distribution of the pixel values (e.g., multiscale histogram, first four moments), and polynomial decomposition of the image. The image transforms that are used are Fourier transform, Chebyshev transform, Wavelet (symlet 5, level 1) transform, and edge transform. A detailed description of the image features can be found in [21, 22]. In this study the larger set of image transforms was used, as described in [21].

After all image features are computed, Fisher scores are computed individually for each image feature, and 93% of the features with the lowest Fisher scores are rejected. The rejection of the 93% of the features was determined experimentally, but as thoroughly discussed in [22, 23], changing this value has marginal effect on the performance. The classification can then be made by using the remaining 7% of the features with a Weighted Nearest Neighbor rule, such that the Fisher scores are used as weights. This type of nonparametric classification can be used not just for finding the class that is the closest to a given test sample, but also to measure the distances between any given test image  to any training image , by using(1)

where  is the Fisher score of feature *f*, and ,  are the values of the feature *f* in the images  and , respectively.

Similarly, the similarity between an image  and a certain class  can be measured using(2)

where  is the computed similarity between the image*m* and the class*c*, *N* is the total number of classes, and  is the shortest weighted Euclidean distance between image*m* and any image in the training set that belongs in the class*c*, as described by (1). This assigns each feature vector of an image with a vector of*N* values within the interval , representing the similarities of the image to each class. Naturally, for classification purposes the class that has the shortest weighted Euclidean distance to a given test image can be determined as the predicted class of that test image. Averaging all similarity vectors of all images of a given class provides a vector that reflects the similarity of that class to each of the other classes. Repeating it for all classes results in a similarity matrix, which represents the similarities between all pairs of classes, and can be also visualized as a phylogeny by using the Phylip package [24]. The analysis of the similarities between classes is described in further detail in [21].

Once each class is assigned with a numeric value, the similarities of any given test image can be used to compute and interpolate a value for that image using(3)

where  is the interpolated value, *N* is the number of classes,  is the similarity of image*i* to the class*c* as determined by (2), and  is the value assigned to class*c*. This interpolation can be used to deduce the values of test images of "in-between'' cases, which do not fit perfectly into a single class.

The classes in this study are the KL grades assigned by readers who manually assessed each of the knee X-rays. A more detailed description of the KL scoring procedure and protocols is described in [10, 25]. As described in Section 1, the KL grades are subjective and dependent on the perception of the individual reader. However, the method described here is based on the assumption that a large set of knees that were all assigned the same KL score provides a reliable representation of that OA stage. This assumption can be used to provide an automated classifier, which can automatically determine the KL grade for a given knee X-ray, and also estimate the KL grade for each test X-ray image with resolution that is higher than the individual KL grades. For instance, an X-ray of an osteoarthritic knee that is between KL grade 2 and KL grade 3 can be assigned with the score of 2.6, rather than classified into KL grade 2 or KL grade 3.

Data for the experiment were X-ray images taken from the BLSA project [26] that were used in [10] and MRI images taken from the Osteoarthritis Initiative [27]. The KL grades of each of the BLSA X-rays were determined by two highly experienced readers, such that a third reader adjudicated knees with discordant grades. Previous studies showed that the classification method used here could automatically classify between KL grade 3 and healthy knees with accuracy of 91%. with sensitivity and specificity of 95% and 86.5%, respectively. KL elements from the Osteoarthritis Initiative (OAI) knees were scored by different readers in each of the four independent centers that participate in the project, and the individual assessments of the KL elements were compiled into a final KL score of the knee.

**Figure 2:**
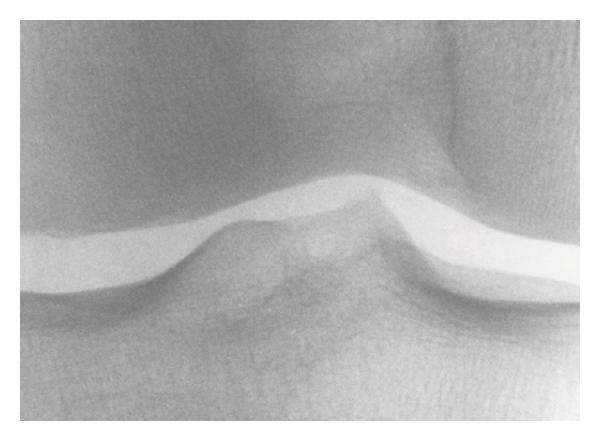
A sample image of a center of a knee X-ray used in this study

**Figure 3:**
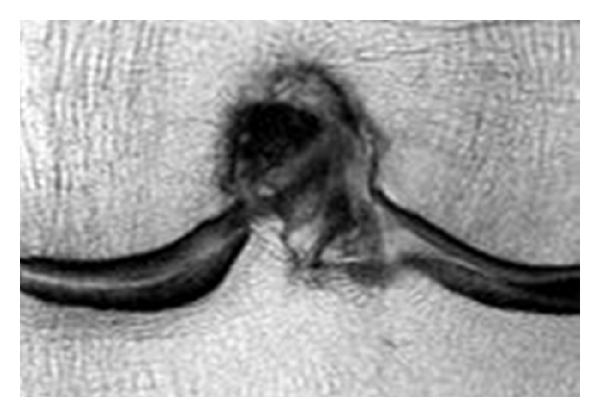
**A sample MRI image used in this study**.

The images were preprocessed such that the center of the joint is detected using the method described in [10], and then the area around the joint center was separated from the image. Figures 2 and 3 show sample X-ray and MRI images used for this study, respectively.

## 3. Results

**Figure 4:**
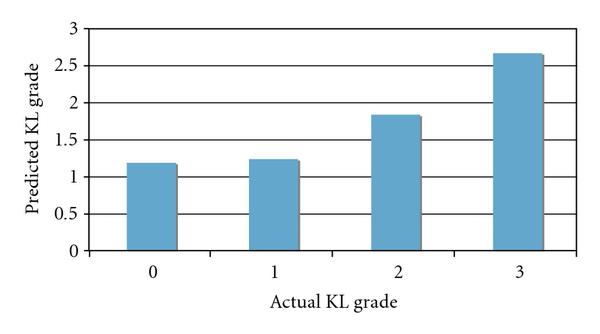
**Predicted KL grade values by the radiographic bone texture using X-rays scored by radiologists**. The figure shows that knees that were scored by radiologists as KL grade 0 are determined by wndchrm to be highly similar to knees scored by radiologists as KL grade 1, indicating that the bone texture does not change significantly between these two grades.

In the experiment with the X-ray images, 30 images of each class were used for training and nine images per class for testing. The experiment was repeated 100 times such that in each run different images were allocated randomly for training and testing. The predicted values of the test images were scored as described in Section 2, and the averaged scores for the different KL grades are shown in Figure 4.

As the figure shows, the mean predicted KL grades of*healthy* and*doubtful* OA joints are nearly identical, showing that there are no substantial differences in the bone texture between healthy joints and*doubtful* OA joints, while significant differences are clearly noticeable for the image analysis of the bone textures of*doubtful* and*mild* OA and*mild* and*moderate* OA. It should be noted that KL grade 1 is determined by the presence of mild osteophytes which are not present in the joint itself and are often difficult to detect using X-ray. KL grade 1 patients usually do not suffer from pain symptoms, but more importantly, are not in the phase of fast loss of cartilage that starts around KL grade 2 and estimated in 0.25 mm per year [28]. While the correlation between the radiographic bone texture and cartilage loss has been identified as described in Section 1, this finding shows that rapid loss of cartilage is accompanied by a faster alteration of the radiographic bone texture, suggesting that both can be related to the same mechanism and become more dominant when OA progresses beyond KL grade 1.

**Figure 5:**
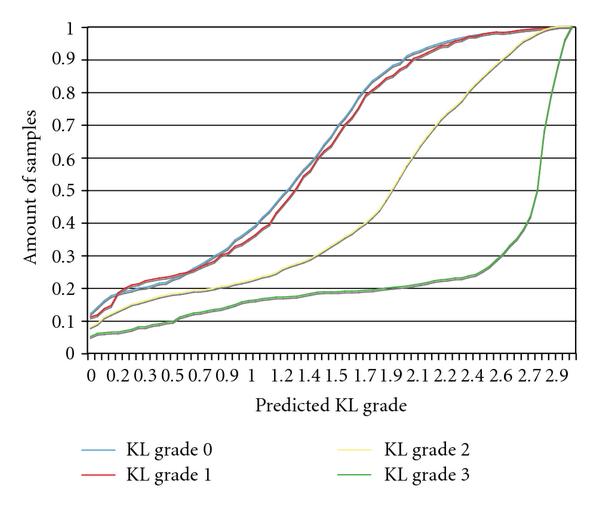
Amount of sample knee X-rays scored lower than a certain predicted KL score (-axis)

Another analysis of the predicted KL grades can be done by using the amount of samples that were predicted to have a KL grade lower than a certain value. Figure 5 shows, for each of the actual KL grades, the amount of sample images (-axis) that their predicted KL grade score is lower than a certain value (-axis). For instance, looking at the curve of the X-rays scored by radiologists as KL grade 3, 0.2 of these X-rays were assigned by the computer analysis with a predicted KL grade lower than 1.9.

As the figure shows, the curves for actual KL grade 0 and actual KL grade 1 are very similar to each other, showing that the distribution of the predicted KL grade values for these two classes is relatively close, while the predicted KL grade distribution is noticeably different for X-rays that were diagnosed by radiologists as KL grades 2 and 3.

**Figure 6:**
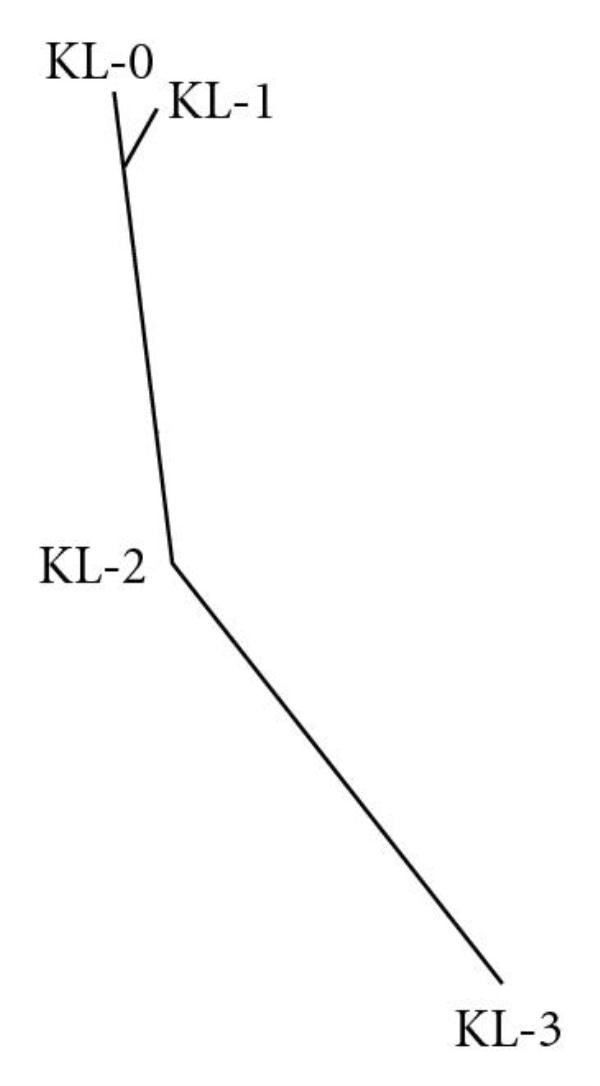
**Evolutionary tree visualizing the similarities between the KL classes, reflected by the image analysis of the bone texture**.

Different visualization of the analysis can be provided by using an evolutionary tree that reflects the similarities between the KL grade classes, as described in Section 2. Figure 6 shows the phylogeny produced by using the Phylip package to visualize the similarity matrix of the KL grade classes.

The phylogeny shows that the system was able to deduce that the KL grade classes represent a continuous process and was also able to find the correct order of the classes. However, it also shows that the X-rays scored as KL grade 0 (healthy joints) are very similar to the X-ray images scored as KL grade 1 (*doubtful* OA), showing that the radiographic texture does not significantly change between these two grades of the disease. This type of visualization can also be useful for more complex experiments, with a larger number of stages and possible paths of progression.

**Figure 7:**
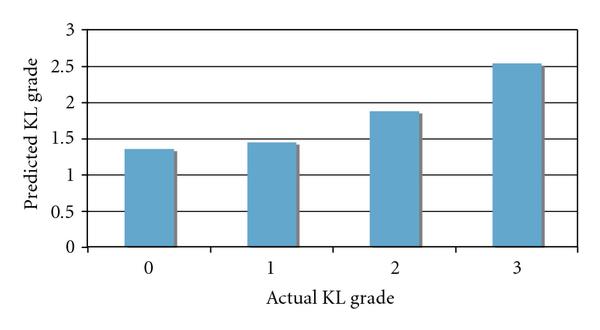
**Predicted KL grade values by the radiographic bone texture using MRI images**. The standard error for all KL grade classes is smaller than 0.1.

A similar experiment with MRI knee images, which features different frequency and different resolution compared to the X-ray images, leads to a similar conclusion. The experiment used 100 knee images (similar to the sample Figure 3) for each KL grade for training and 50 images per class for testing. The experiment was repeated 20 times such that in each run different images were used for training and testing. The averaged predicted values of the test images are shown in Figure 7.

As before, this shows that the progression of the alteration of the bone texture shows no significant difference between healthy (KL grade 0) and*doubtful* OA (KL grade 1) joints, and a faster process of bone alteration starts when the disease reaches KL grade 2. The fact that the image analysis method does not detect the differences between healthy and *doubtful* OA joints shows that the bone texture does not change significantly between these two grades, compared to the alteration of the bone texture associated with further stages of the disease.

## 4. Discussion

While osteoarthritis is one of the most prevalent diseases in the industrialized world, and one of the most significant economical burdens on the healthcare system, little has yet been discovered about its physiological mechanisms, which their understanding is acute for the purpose of developing drugs or preventive treatments. For instance, it is yet unknown whether OA is a systemic clinical condition driven by controlled physiological pathways or simply stochastic "wear and tear'' that leads to cartilage degeneration [3].

In this study we used a quantitative comparison of X-ray images to analyze the progression of OA and the differences between the KL grades. The images are compared by using a large set of image content descriptors computed from each X-ray, and the weighted Euclidean distance reflects the similarity between each two X-rays in the dataset. This quantitative assessment of the differences between images is used to quantify the differences between the KL grades, which are determined quantitatively by averaging a large number of images from each grade and providing an*average* distance between each two neighboring grades based on the physiology of the knee.

The quantitative analysis of the differences between the different KL grades shows that the alteration of the bone texture, which has been associated with OA as discussed in Section 1, does not progress at a constant rate, which can be interpreted as an indication that OA is not driven solely by stochastic accumulation of damage and that controlled physiological processes can be involved in the processes of cartilage degeneration. It also shows that the progression of the biochemical and biomechanical processes associated with the disease progress faster between KL grade 1 and KL grade 2. These findings suggest that the KL scale might not be linear to the actual progression of OA and also indicate that understanding the mechanisms of the disease might require the analysis of changes between KL grade 1 to KL grade 2.

While it can be assumed that the image analysis method might not be sensitive enough for detecting the differences between healthy and *doubtful* OA joints, it should be noted that the same method is sensitive enough to detect osteoarthritis by examining the radiographic texture of the bone  years before the disease becomes symptomatic [25], and when the joints are still scored by radiologists as healthy.

The "gold standard'' for this study is the KL grades, which can be subjective, and not necessarily an accurate quantitative reflection of the actual progression of OA. Since the classification between*doubtful OA* (KL grade 1) and nonosteoarthritic joints can be difficult, the findings presented in Section 3 might be due to the difficulties of the readers to distinguish between these two grades.

The KL classification scheme is exposed to the subjectiveness of the reader, and it can not be considered as an accurate quantitative method for OA assessment. Since computer analysis of the texture can provide an objective measurement, it can ultimately provide a systematic and accurate scoring system for the evaluation of the presence or progression of OA. This scoring technique can better reflect the actual progress of the disease, and its quantitative fashion can potentially make it suitable for studying the disease mechanisms or the effect of drugs and treatments on the clinical condition. While indicators such as the KL grade or pain symptoms are merely rough measurements of the disease progression, the computer analysis of the bone texture can provide a higher resolution estimation of the state of the disease, and its response to drugs can be more sensitive and objective than other OA indicators.

In this study we apply the*wndchrm* tool to the assessment of the progression of OA, but it should be noted that*wndchrm* is a general-purpose biomedical image analysis tool and can provide an informative analysis for a variety of subjects, magnifications, and types of imaging [29]. Therefore, we argue that it is possible that the image analysis method described in this paper can be used for studying and evaluating the progression of other continuous clinical conditions that can be sensed and diagnosed by using microscopy or other forms of biomedical imaging. Medical imaging is used for diagnosis and prognosis of very many diseases and clinical conditions, and the link between image morphology and disease progression has been well established. In this paper we propose to use image analysis to detect stages of rapid progression of the disease, which can be used to better study the disease mechanisms.

Finally, while this study focused on an unbiased analysis of the image content, a more comprehensive study can also include clinical data such as pain, history of injuries, relevant medication information, age, weight, BMI, and other clinical indicators that can be relevant to OA. These data can provide a more detailed analysis that can detect stages in the progression of the disease that correspond to other clinical data that affect osteoarthritis.

The software used for the experiments described in this paper, including the source code, is available for free download at http://www.phy.mtu.edu/~lshamir/downloads/ImageClassifier, and users are encouraged to download and use it for the analysis of continuous image data of disease progression. Technical description of the code is available at [21].
